# Infusion of Autologous Retrodifferentiated Stem Cells into Patients with Beta-Thalassemia

**DOI:** 10.1100/tsw.2006.229

**Published:** 2006-10-09

**Authors:** Ilham Saleh Abuljadayel, Tasnim Ahsan, Huma Quereshi, Shakil Rizvi, Tamseela Ahmed, Sabiha Mirza Khan, Jawaid Akhtar, Ghazi Dhoot

**Affiliations:** ^1^ TriStem U.K. Limited, Karachi, Pakistan; ^2^ Jinnah Postgraduate Medical Centre, Karachi, Pakistan; ^3^ Pakistan Medical Research Council, Islamabad, Pakistan; ^4^ Kharadar General Hospital, Karachi, Pakistan; ^5^ National Medical Centre, Karachi, Pakistan; ^6^ Orthopedic and Medical Institute, Karachi, Pakistan

**Keywords:** Stem Cell Therapy, Retrodifferentiation, Somatic Cell Reprogramming or Plasticity, Beta-Thalassemia, Iron Overload, Serum Ferritin, Anaemia, Fetal Hemoglobin

## Abstract

Beta-thalassemia is a genetic, red blood cell disorder affecting the beta-globin chain of the adult hemoglobin gene. This results in excess accumulation of unpaired alpha-chain gene products leading to reduced red blood cell life span and the development of severe anemia. Current treatment of this disease involves regular blood transfusion and adjunct chelation therapy to lower blood transfusion–induced iron overload. Fetal hemoglobin switching agents have been proposed to treat genetic blood disorders, such as sickle cell anemia and beta-thalassemia, in an effort to compensate for the dysfunctional form of the beta-globin chain in adult hemoglobin. The rationale behind this approach is to pair the excess normal alpha-globin chain with the alternative fetal gamma-chain to promote red blood cell survival and ameliorate the anemia. Reprogramming of differentiation in intact, mature, adult white blood cells in response to inclusion of monoclonal antibody CR3/43 has been described. This form of retrograde development has been termed “retrodifferentiation”, with the ability to re-express a variety of stem cell markers in a heterogeneous population of white blood cells. This form of reprogramming, or reontogeny, to a more pluripotent stem cell state ought to recapitulate early hematopoiesis and facilitate expression of a fetal and/or adult program of hemoglobin synthesis or regeneration on infusion and subsequent redifferentiation. Herein, the outcome of infusion of autologous retrodifferentiated stem cells (RSC) into 21 patients with beta-thalassemia is described. Over 6 months, Infusion of 3-h autologous RSC subjected to hematopoietic-conducive conditions into patients with beta-thalassemia reduced mean blood transfusion requirement, increased mean fetal hemoglobin synthesis, and significantly lowered mean serum ferritin. This was always accompanied by an increase in mean corpuscular volume (MCV), mean corpuscular hemoglobin (MCH), and mean corpuscular hemoglobin concentration (MCHC) in such patients. No adverse side effects in response to the infusion of autologous RSC were noted.This novel clinical procedure may profoundly modify the devastating course of many genetic disorders in an autologous setting, thus paving the way to harnessing pluripotency from differentiated cells to regenerate transiently an otherwise genetically degenerate tissue such as thalassemic blood.

### INTRODUCTION

The homozygous, beta-globin chain mutation in the adult hemoglobin (Hb A) gene gives rise to the blood disorder beta-thalassemia major. In this genetic disorder, red blood cells have a short life span due to excessive accumulation of the unpaired alpha-globin chain[1,2]. Possessing no single, stable, molecular configuration, alpha-hemoglobin aggregates and precipitates in early hemoglobin-producing cells in the bone marrow, which leads to apoptosis of these cells and ineffective erythropoiesis The red cells that reach the peripheral blood also contain excess alpha-globin; this causes the formation of inclusion bodies and an increase in reactive oxygen species levels, which leads to membrane damage and causes these cells to hemolyze preferentially. Beta-thalassemia major is treated with regular blood transfusion and concomitant administration of chelating agents[3] to reduce red blood transfusion–induced hemochromatosis. Adequate chelation of such patients reduces iron accumulation and prevents organ damage, resulting in a consistent decrease of morbidity and mortality.

On the other hand, the realization that increased fetal hemoglobin (Hb F) production in patients with beta-thalassemia, sickle cell disease, and hereditary persistence of fetal hemoglobin result in a much milder anemia intensified interest in finding an alternative approach to treat these disorders[4]. The uses of a variety of drugs that have gamma-globin chain–inducing activity, such as 5-azacytidine, hydroxyurea, erythropoietin, butyrate derivatives, and combinations of these drugs, have been explored[5,6,7,8] .

It is known that augmented postnatal expression of Hb F by using a variety of drugs, such as 5-azacytidine, hydroxyurea, erythropoietin, and butyrate derivatives, is perhaps the most widely recognized modulator of beta-thalassemia and sickle cell disease severity. For example, hydroxyurea decreases mature bone marrow cells and increases erythroid regeneration by less mature cells[9], which produce Hb F. Erythropoietin results in an increase of new red cell production[10]. 5-Azacytidine causes an increased hypomethylation of gamma gene promoters, increasing expression and Hb F[11]. Butyric acid results in the inhibition of histone deacetylase[12], changing chromatin structure, thus elevating Hb F expression in which the gamma-globin subunit replaces the mutated beta-globin subunit in the hemoglobin tetramer so that, eventually, Hb F replaces Hb A as the primary component of red cells.

Potentially, somatic cell reprogramming technologies or plasticity[13] induced in more committed adult stem cells may offer novel solutions to manage a variety of hematological and degenerative diseases. One example, the hypothesis that intact, mature white blood cells can be reprogrammed to become pluripotent stem cells via a process of retrodifferentiation[14,15,16], ought to reswitch earlier programs of hematopoiesis. For example, reversion of beta-thalassemic leukocytes to a primitive stage in hematopoiesis ought to reactivate genes that are associated with early erythropoiesis[17], at least at a fetal stage, and allow the expression of Hb F instead of Hb A on redifferentiation. This stepwise reversion of differentiation may lead to amelioration of the severity of the anemia and the incorporation of an otherwise injurious, as well as toxic, metal such as iron into more useful heme synthesis[18].

Herein, we tested the safety and efficacy of infusing 3-h autologous retrodifferentiated stem cells (RSC) into patients with beta-thalassemia.

## MATERIALS AND METHODS

### Patients

The study was designed according to the standards of Good Clinical Practice and approved by the Hospital Ethics Committee of Orthopedic Medical Institute (OMI), Karachi, in joint consultation with the Pakistani Medical Research Council. Informed consent from all study participants was obtained by Drs. SR, TA, and TA. The selection criteria were as detailed in Table 1. Enrolled into the study were 19, 1, and 1 patients with beta-thalassemia major, intermedia (beta-thalassemia/Hb E variant), and thalassemia/sickle cell anemia (beta-thalassemia/SC), respectively. Details of all patients who entered the study are shown in Table 2. All patients received Desferal prior to the study as a chelating agent, but all were noncompliant due to shortage of supply or noncontinuous administration of the drug. None of these patients were on Hb F-augmenting agents. Only 1 thalassemic patient was splenectomized (patient 11). All patients who entered the study were recently transfused, apart from 1 (patient 21). All patients, apart from 2, had beta-thalassemia major and were receiving blood transfusion about every 1–3 weeks. Only 1 patient (beta-thalassemia/SC) never received a blood transfusion prior to the procedure (patient 21). Patient weight, height, spleen, and liver size, as well as blood transfusion requirement, were determined prior to and following infusion of the RSC. Blood transfusion threshold was set at 7 g/dl due to the severe shortage of blood supply. Blood products were obtained from various blood banks in the city. Screened, packed red blood cells were irradiated prior to transfusion. None of these patients received irradiated blood prior this study. Transfusion requirement was expressed as the number of packed red blood cells (each unit consisted of 250 ml of packed red blood cells) transfused every 3 months pre- and postinfusion. These thalassemic patients will be monitored for 2 years following infusion of the RSC. All clinical monitoring of patients were carried out by Drs. SR, TA, and TA.

**TABLE 1**
**Selection Criteria**

| |
|---|
| Patient has beta-thalassemia, is anemic, and requires regular transfusions |
| Patient at least 3 years old and body weight > 10 kg |
| Patient not on any antibiotics |
| Patient not receiving Hb F-enhancing agents |
| Patient did not receive prior immunosuppressive therapy < 1 month ago |
| Patient negative for HIV, HCV (serology or PCR), CMV (IgM), and HBsAg infections |

### Preparation and Infusion of Retrodifferentiated Stem Cells

The RSC were prepared under hematopoietic-conducive condition (HCC) by TriStem Corporation at the study site and as described previously[14] with the following modifications. Using double-lumen or single-lumen jugular catheterization set (Arrow International) as an input line and under local anesthesia, a central venous line was inserted into patients via the jugular vein on the day of the procedures. The insertion of the central venous lines was performed by Dr. JA. For output line, an intravenous antecubital fossa line (VASOCAN BRAUNULE) was inserted. Patients were apheresed by representatives from Gambro BCT in Pakistan; 1.5 to 2 total blood volume was apheresed using The Cobe Spectra device and the white blood cell collection kit (both from Gambro BCT). A total of 150–200 ml of Buffy coats were collected. Cells were retrodifferentiated under aseptic conditions within the white blood cell bag by adding 30 ml of Iscove's Modified Dulbecco's Medium (Invitrogen) and 1000 µg of highly purified CR3/43 monoclonal antibody (specially prepared by DakoCytomation under good tissue culture practice). This monoclonal antibody is raised against the homologous region of the beta-chain of the Major

Histocompatibility Complex (MHC) class II antigens. Thereafter, cells were mixed and incubated at 37°C in a tissue culture incubator for 3-h. On completion of the retrodifferentiated reaction, an aliquot of cells was collected aseptically by sealing a portion of the tubing containing the cell suspension leading to the white blood cell bag for examination. Prior to infusion, the RSC were washed in equal volume of saline solution and resuspended in half the volume in the same solution following an 10-min agitation cycle using the COBE 2991 Cell processor device and associated kit (both from Gambro BCT). Following washing, cells were infused immediately into patients via the jugular vein under gravity.

**TABLE 2**
**Clinical and RSC Infusion Details as well as Outcome of Patients with Beta-Thalassemia Major (TM), Thalassemia/Hb E Intermedia (T/Hb E I), and Thalassemia/Sickle Cell Disease (T/SC)***

| Patient | Sex | Age/ Year | Anti-HCV | Disease | Nucleated Cells Infused $10^6$/kg | CD34 Cells Infused $10^6$/kg | Significant Clinical Events | Mortality and Safety |
|---|---|---|---|---|---|---|---|---|
| 001 | M | 7 | –ve | TM | 180.05 | 121.5 | Malaria | Alive |
| 002 | F | 8 | –ve | TM | 199.75 | 134.8 | | Alive |
| 003 | F | 10 | –ve | TM | 80.03 | 54 | | Alive |
| 004 | F | 10 | –ve | TM | 196.98 | 132.9 | Typhoid | Alive |
| 005 | F | 13 | –ve | TM | 143.39 | 96.5 | | Alive |
| 006 | M | 13 | B +ve | TM | 460.53 | 257.9 | | Alive |
| 007 | F | 8 | –ve | TM | 292.88 | 197.6 | | Alive |
| 008 | F | 4 | –ve | TM | 388.91 | 262.5 | | Alive |
| 009 | M | 8 | B +ve | TM | 148.9 | 100.5 | *E. coli*-infected transfusion | Died after 3 months |
| $010^{\pm o}$ | M | 17 | B +ve | TM | 129.76 | 87.6 | | Alive lost to follow-up after 2 weeks |
| 011 | F | 10 | B +ve | TM | 323.13 | 218 | | Alive |
| 012 | M | 4 | –ve | TM | 342.99 | 231.5 | | Alive |
| $013^{\pm}$ | M | 6 | –ve | TM | 343.27 | 231.5 | | Alive |
| 014 | M | 8 | B +ve | TM | 101.7 | 68.6 | Cholera | Alive |
| 015 | M | 10 | +ve | TM | 111 | 74.9 | | |
| $016^{\pm o \varphi}$ | F | 16 | +ve | TM | 30.01 | 20.25 | Cholera and PAH | Died after 2 months |
| 017 | F | 9 | –ve | TM | 158.44 | 106.9 | | Alive |
| 018 | M | 35 | –ve | T/Hb E I | 26.99 | 18.2 | | Alive |
| 019 | M | 7 | +ve | TM | 126.78 | 85.6 | Cholera | Alive |
| $020^{o}$ | M | 5 | –ve | TM | 485.42 | 327.7 | | Alive |
| 021 | M | 18 | –ve | T/SC | 356 | 285 | | Alive |

* Patients positive or borderline positive (B +ve) were negative for HCV infection by PCR. No patient experienced any adverse side effects due to RSC infusion as determined by vital sign monitoring, karyotyping/G-banding, and echocardiogram analysis. Superscript symbols $^{o}$ and ± denote no bone marrow or karyotyping and G-banding analysis performed, respectively. Symbol φ denotes abnormal echocardiogram analysis.

### Methods and Analysis

#### CD34 Enumeration by Flow Cytometery

Prior to infusion of the 3-h RSC into patients, CD34-positive cells were enumerated using FACScan or FACScalibur systems, Beckton Dickinson (BD). Briefly, 1 million cells were stained with antibodies against human CD34-PE-Cy5, CD38-FITC, and CD45-APC or FITC (DakoCyomation and BD) and CD3-PE-Cy5. Cells were also stained with the relevant isotype controls. Cells having forward and side scatter typical of mononuclear cells were gated, and the absolute number of CD34-positive cells was determined by multiplying absolute lymphocyte count obtained by the CBC counter by the percentage of CD34-positive cells in lymphocytes gate.

#### Virological, Hematological, and Biochemical Monitoring of Patients

All patients and blood product were screened for CMV (IgM), HIV, HBsAg, HCV (Abbot AXSYM system, ABBOT Diagnostics), and malaria infection. If the HCV serological test was positive or borderline, this was reconfirmed by PCR. Differential, complete white blood cell count (CBC) was performed (Sysmex XT-1800 i) on patient's blood on screening, prior to apheresis, immediately following apheresis, and 1, 2, 3 days, 2 weeks, 1, 2, 3, and 6 months following infusion of the RSC. At the same time, reticulocyte count was performed manually using methylene blue. Serum ferritin (Elecsys Ferritin reagent kit, Roche Diagnostics GmbH and Elecsys module E 170), alanine aminotransferase (ALT), aspartate aminotransferase (AST), lactate dehydrogenase (LDH), creatinine, bilirubin (all Ecoline, Merck Diagnostica), total protein, and albumin (Bioscience) were determined at baseline, 1, 2 weeks, 1, 2, 3, and 6 months postinfusion. Echocardiogram and bone density analysis were performed on all patients 2–3 month postinfusion of RSC. These tests were performed at the study site, OMI Hospital, Karachi. At the same time points, hemoglobin variants, including Hb F (Bio-Rad Variant), were determined using high-performance liquid chromatography (HPLC). This was performed in conjunction with measuring hemoglobin indices using the Coluter STK. Karyotyping and G-banding were performed on all patients before and 3–6 months following the infusion of the RSC. Both tests, including the reconfirmation of HCV infection, were performed at the Aga Khan University Hospital. Following patient consent, the same center performed bone marrow smear and trephine section analysis of thalassemic patients around 2 weeks postinfusion of the RSC. Blood transfusion requirements were monitored over the study period and compared to baseline levels prior to infusion of the RSC. In-house and laboratory independent testing was coordinated and overseen by Dr. SMK.

#### Statistics

The significance of all laboratory data was analyzed by determining the difference between before and after infusion of the RSC using the Wilcoxon paired test (MedCalc software). Data were considered significant when $p$ value was $<0.05$. Results were expressed as the mean ± SEM, range, and number of samples measured at specified time point.

### RESULTS

### Patients and Cells Infusate

The absolute number of nucleated and CD34 cells infused was calculated and is presented in Table 2. A representative FACScan plot showing the immunophenotype of the infused RSC subjected to 3-h HCC is shown in Fig. 1. All patients tolerated the RSC without any side effects. All patients were discharged on the second day postinfusion. Karyotyping and G-banding studies were normal in all patients before and after 3–6 months postinfusion of RSC (data not shown).

One patient was lost in the follow-up 3 weeks (patient 10, Table 2) following infusion of the RSC, but is reported to be alive at the time of writing. Another (patient 9, Table 2) died after 3 months from blood transfusion–related complications. Of four patients that were siblings (patients 14, 15, 16, and 19, Table 2), two contracted cholera poststudy (patients 14 and 16, Table 2). On treatment, one recovered (patient 14, Table 2) and one died 2 months postinfusion (patient 16, Table 2). This female patient was also

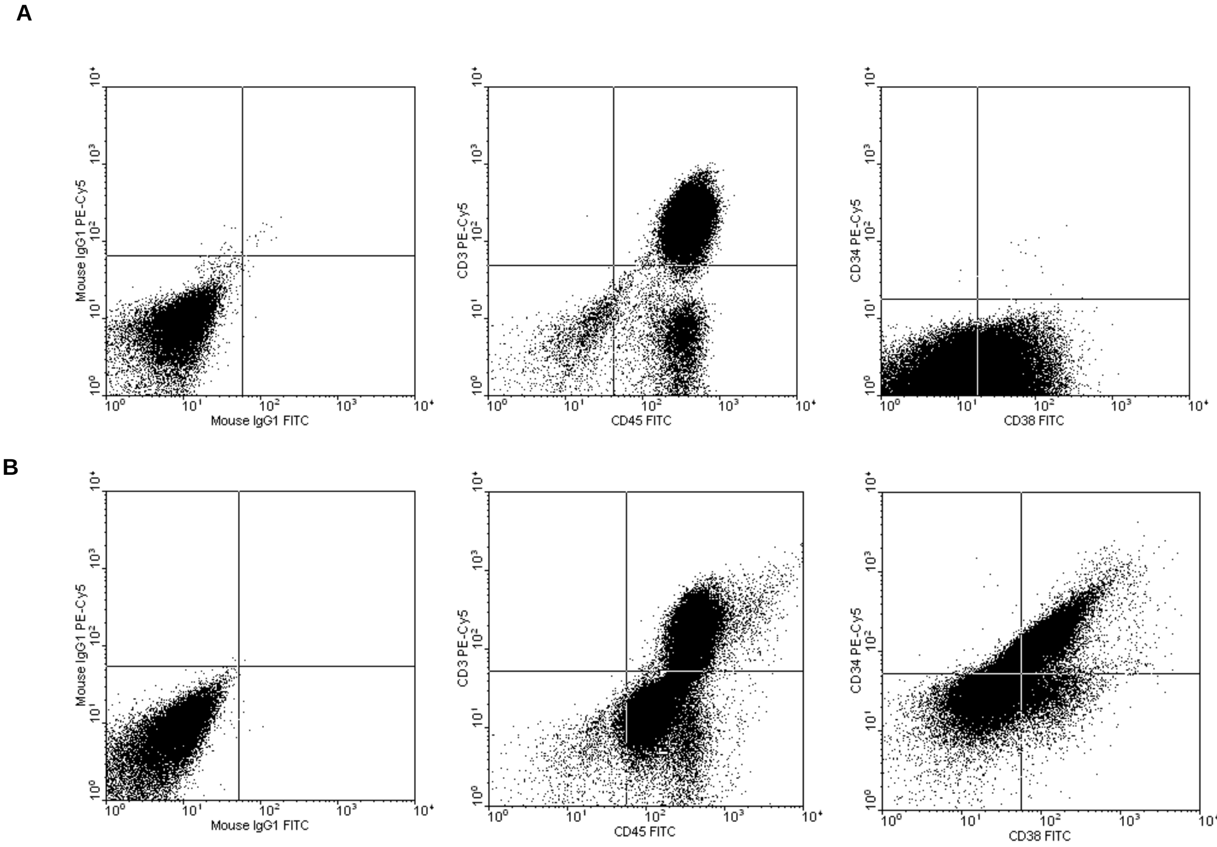

**FIGURE 1.** Representative immunophenotyping plots of apheresed mononuclear cells (Buffy coat) of patients with beta-thalassemia prior (A) and following (B) 3-h exposure to HCC. The panels on the left represent isotype controls (mouse IgG1-FITC and PE-Cy5 conjugates). The panels in the middle and right show costaining for CD45-FITC and CD3-PE-Cy5, and CD38-FITC and CD34-PE-Cy5, respectively.

suffering from acute pulmonary artery hypertension (PAH) as measured by echocardiogram. One patient had malaria falciparum (patient 1, Table 2), which resolved on prophylactic treatment. One patient (patient 4, Table 2) developed typhoid fever postinfusion and was treated successfully. All patients reported 50–75% improvement in physical activity and well-being following infusion of the RSC. The mean weight of patients showed a statistically significant increase ($p$ = 0.01) at 6 months postinfusion of the RSC when compared to baseline (Table 3). The mean height of patients showed a statistically significant increase at 3 (0.0001) and 6 (0.0001) months postinfusion when compared to baseline (Table 3). Furthermore, blood transfusion requirement dropped from a mean 6 ± 0.7 units packed red blood cells/90 days at baseline to 4 ± 0.7 ($p$ = 0.01) and 3 ± 0.6 ($p$ = 0.001) units of packed cells/90 days at 3 and 6 months, respectively, which was statistically significant when compared to baseline (Table 3). Only two patients, one with thalassemia intermedia (beta-thalassemia/Hb E) and the other with thalassemia/sickle cell anemia, are transfusion independent 6 months following receipt of the RCS (Table 2, patients 18 and 21, respectively, and Figs. 2 and 3, respectively).

### Fetal Hemoglobin and Red Blood Cell Indices

The mean absolute amount of Hb F increased to a statistically significant level at 1 ($p = 0.02$), 2 ($p = 0.01$), 3 ($p = 0.002$), and 6 ($p = 0.002$) months postinfusion of the RSC when compared to baseline level (Table 3, Fig. 4). This was always accompanied by a significant decrease in Hb A (Table 3, Fig. 4) at 1 ($p = 0.01$), 2 ($p = 0.002$), 3 ($p = 0.002$), and 6 ($p = 0.001$) months postinfusion when compared to baseline. Mean

**TABLE 3**
**Mean Weight, Height, Absolute Hb F, Transfusion Requirement, %Hb A, %Hb A2, MCV, MCH, MCHC, Serum Ferritin, LDH, SGOT, and SGPT Before and After RSC Infusion into Patients with Beta-Thalassemia***

| Test | Baseline | 1 Week | 2 Weeks | 1 Month | 2 Months | 3 Months | 6 Months |
|---|---|---|---|---|---|---|---|
| Weight/kg | 24.3 ± 2.1<br>(12–50)<br>N = 21 | ND | ND | ND | ND | 23.7 ± 2.2<br>(13–51)<br>P = 0.1<br>N = 19 | 25.2 ± 2.5<br>(15–52)<br>P = 0.01<br>N = 17 |
| Height/cm | 123.1 ± 4.4<br>(94–170)<br>N = 21 | ND | ND | ND | ND | 124.4 ± 4.7<br>(95–170)<br>P = 0.0001<br>N = 19 | 127.4 ± 4.8<br>(98–170)<br>P = 0.0001<br>N = 17 |
| Hb F g/L | 1.2 ± 0.6<br>(0–8.8)<br>N = 20 | 1.1 ± 0.5<br>(0–8.7)<br>P = 0.6<br>N = 20 | 1.5 ± 0.6<br>(0–8.4)<br>P = 0.9<br>N = 19 | 2.9 ± 1<br>(0–10.7)<br>P = 0.02<br>N = 16 | 3 ± 1.2<br>(0–21.7)<br>P = 0.01<br>N = 18 | 3.9 ± 1.5<br>(0–27)<br>P = 0.002<br>N = 18 | 4 ± 1.6<br>(0–27.9)<br>P = 0.002<br>N = 17 |
| Transfusion requirement; packed red blood cells (250 ml)/90 days | 6 ± 0.7<br>units/90 days<br>(0–12)<br>N = 21 | | | | | 4 ± 0.7<br>units/90 days<br>(0–14)<br>P = 0.01<br>N = 19 | 3 ± 0.6<br>units/90 days<br>(0–11)<br>P = 0.001<br>N = 18 |
| %Hb A | 89 ± 5.8<br>(0–98)<br>N = 18 | 91.3 ± 4.8<br>(0–98)<br>P = 0.3<br>N = 17 | 88.2 ± 5.5<br>(0–98)<br>P = 0.1<br>N = 16 | 84.8 ± 6.6<br>(0–98)<br>P = 0.01<br>N = 14 | 89.5 ± 3.9<br>(26.6–98)<br>P = 0.002<br>N = 16 | 82.6 ± 7<br>(0–98)<br>P = 0.001<br>N = 16 | 80.6 ± 7<br>(0–98)<br>P = 0.001<br>N = 15 |
| %Hb A2 | 2.87 ± 0.3<br>(0–5.3)<br>N = 17 | 2.93 ± 0.2<br>(0–5.3)<br>P = 0.3<br>N = 15 | 2.97 ± 0.2<br>(0–4.9)<br>P = 0.2<br>N = 14 | 3.04 ± 0.3<br>(0–5.5)<br>P = 0.05<br>N = 12 | 2.79 ± 0.2<br>(0–5)<br>P = 0.1<br>N = 14 | 2.89 ± 0.3<br>(0–5.9)<br>P = 0.2<br>N = 15 | 2.82 ± 0.2<br>(0–5.6)<br>P = 0.2<br>N = 14 |
| MCV<br>fl | 75.2 ± 1.6<br>(58–86)<br>N = 21 | 78.0 ± 1.7<br>(57–88)<br>P = 0.003<br>N = 20 | 78.6 ± 1.6<br>(60–89)<br>P = 0.002<br>N = 21 | 79.4 ± 1.6<br>(60–89)<br>P = 0.001<br>N = 21 | 82 ± 1.6<br>(57–89)<br>P = 0.005<br>N = 19 | 80 ± 2<br>(51–92)<br>P = 0.01<br>N = 19 | 79 ± 1.9<br>(56–88)<br>P = 0.06<br>P* = 0.01<br>N = 18 |
| MCH<br>Pg | 24.6 ± 0.5<br>(19–28)<br>N = 21 | 25.6 ± 0.6<br>(19–29)<br>P = 0.01<br>N = 20 | 25.6 ± 0.6<br>(19–29)<br>P = 0.004<br>N = 21 | 26.4 ± 0.6<br>(20–31)<br>P = 0.0004<br>N = 21 | 27.2 ± 0.5<br>(19–30)<br>P = 0.001<br>N = 20 | 26.6 ± 0.7<br>(18–30)<br>P = 0.005<br>N = 19 | 27.3 ± 0.7<br>(18–30)<br>P = 0.002<br>N = 18 |
| MCHC<br>% | 32.7 ± 0.2<br>(31–34)<br>N = 20 | 32.9 ± 0.2<br>(32–35)<br>P = 0.4<br>N = 19 | 32.5 ± 0.2<br>(31–34)<br>P = 0.3<br>N = 20 | 33.3 ± 0.3<br>(31–36)<br>P = 0.04<br>N = 20 | 33.5 ± 0.3<br>(30–36)<br>P = 0.01<br>N = 18 | 33.5 ± 0.2<br>(32–35)<br>P = 0.002<br>N = 20 | 34.5 ± 0.2<br>(33–36)<br>P = 0.0001<br>N = 19 |
| Serum Ferritin ng/ml | 4463 ± 598<br>(197–9012)<br>N = 21 | 6029 ± 1255<br>(135–23800)<br>P = 0.6<br>N = 20 | 5008 ± 860<br>(315–17770)<br>P = 0.8<br>N = 21 | 4325 ± 584<br>(114–8436)<br>P = 0.9<br>N = 19 | 3800 ± 480<br>(1068–7985)<br>P = 0.07<br>N = 19 | 4052 ± 655<br>(36.9–9210)<br>P = 0.1<br>N = 18 | 3069 ± 495<br>(46–9094)<br>P = 0.001<br>N = 18 |
| LDH | 622.2 ± 120 | 738.5 ± 132 | 934 ± 240 | 826.2 ± 89 | 943.5 ± 141 | 1069.6 ± 396 | 856.8 ± 101 |

| | | | | | | | |
|---|---|---|---|---|---|---|---|
| | (177–2650) | (187–2552) | (164–4678) | (101–1545) | (339–2300) | (277–7600) | (354–1919) |
| U/L | N = 21 | P = 0.1 | P = 0.15 | P = 0.01 | P = 0.04 | P = 0.0.03 | P = 0.02 |
| | | N = 20 | N = 21 | N = 18 | N = 19 | N = 18 | N = 18 |
| SGOT | 73 ± 12 | 138.3 ± 32 | 112.9 ± 30 | 104.3 ± 27 | 92.2 ± 17 | 118.2 ± 24 | 97.5 ± 19 |
| | (11–241) | (11–491) | (13–560) | (14–491) | (29–356) | (22–377) | (17–364) |
| U/L | N = 21 | P = 0.001 | P = 0.05 | P = 0.06 | P = 0.07 | P = 0.005 | P = 0.15 |
| | | N = 20 | N = 21 | N = 18 | N = 19 | N = 19 | N = 18 |
| SGPT | 99.3 ± 20 | 172.5 ± 41 | 128.2 ± 33 | 116.5 ± 31 | 102 ± 22 | 124.3 ± 28 | 93.5 ± 18 |
| | (17–382) | (12–640) | (21–584) | (11–580) | (25–470) | (14–462) | (16–297) |
| U/L | N = 21 | P = 0.03 | P = 0.2 | P = 0.4 | P = 0.6 | P = 0.5 | P = 0.3 |
| | | N = 20 | N = 21 | N = 18 | N = 19 | N = 19 | N = 18 |

* Values in brackets denote range and N denotes number of patients in each group. P denotes *p* value and P* denotes *p* value analysis without one outlier (patient with thalassemia Hb E disease).

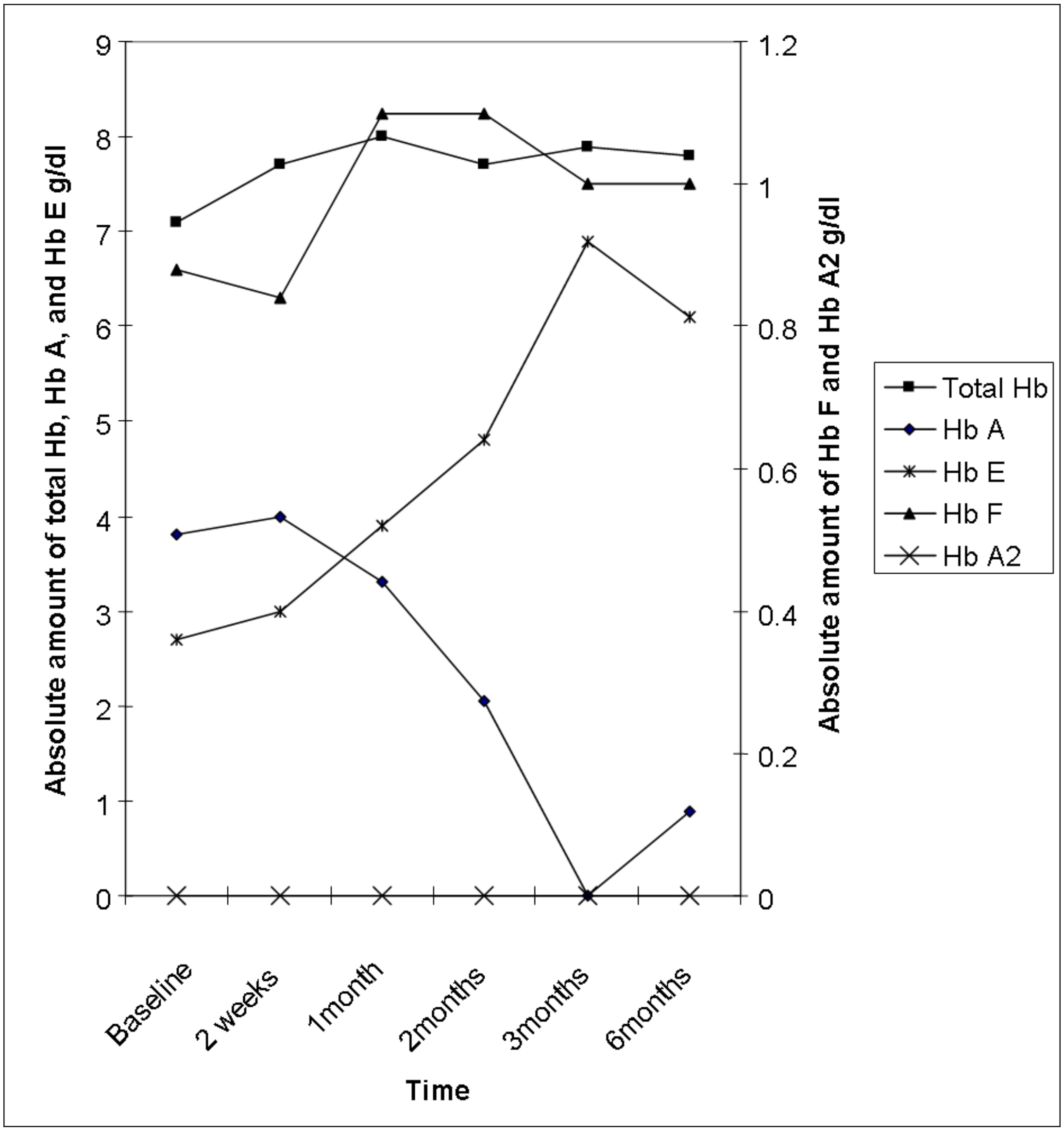

**FIGURE 2.** Hemoglobin type change at 2 weeks, 1, 2, 3, and 6 months postinfusion of RSC into a patient with beta-thalassemia/Hb E (intermedia) disease when compared to baseline. This patient became transfusion independent postinfusion of the RSC.

absolute Hb A2 increased to a borderline, nonstatistically significant level (*p* = 0.05) at 1 month postinfusion when compared to baseline (Table 3, Fig. 4). Thereafter, mean Hb A2 remained more or less at the same levels as baseline. The general trend of Hb F switching appears to increase with time (Table 3, Fig. 4). These were accompanied by a statistically significant increase in mean corpuscular volume (MCV) and mean corpuscular hemoglobin (MCH) at all time points measured when compared to baseline level (Table 3, Figs. 5 and 6). In consideration, all patients entering the study had artificially high MCV and MCH at baseline due to recent blood transfusions. Mean mean corpuscular hemoglobin concentration (MCHC) rose to statistically significant levels at 1 (*p* = 0.04), 2 (*p* = 0.01), 3 (*p* = 0.002), and 6 (*p* = 0.0001) months postinfusion when compared to baseline (Table 3, Fig. 7).

Interestingly, one patient (patient 18, Fig. 2) with beta-thalassemia intermedia (beta thalassemia/Hb E) who is transfusion independent showed a significant decrease in Hb A (4.2 g/dl baseline). This patient's Hb A reached zero at 3 months postinfusion with concomitant increase in Hb F (1.1 g/dl) and Hb E (7.4 g/dl) when compared to baseline levels (Hb F = 0.77 g/dl, Hb E =3.1 g/dl). At 6 months postinfusion, the Hb A of this patient reswitched to a level of 0.9 g/dl with Hb F and Hb E at 0.9 g/dl and 6 g/dl, respectively. This

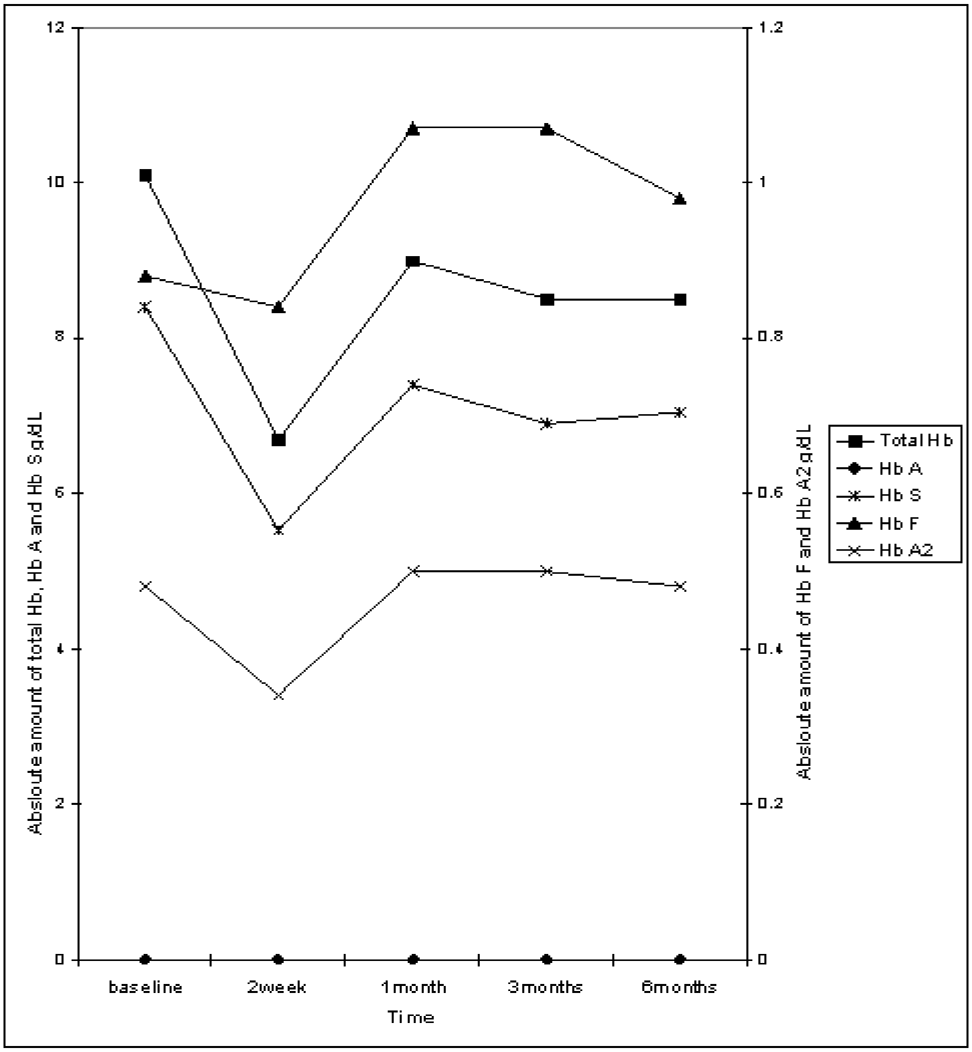

**FIGURE 3**. Hemoglobin type changes at 2 weeks, 1, 3, and 6 months postinfusion of the RSC into a patient with beta-thalassemia/sickle cell disease when compared with baseline. This patient never received a blood transfusion pre- or postinfusion of the RSC.

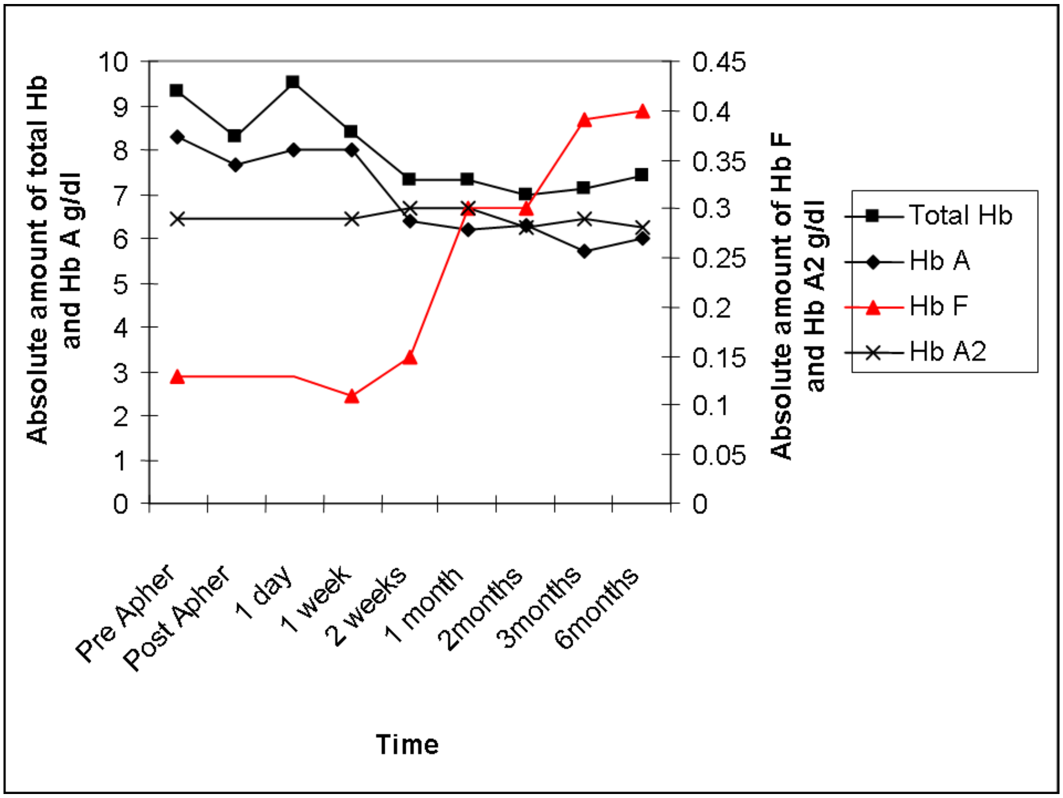

**FIGURE 4.** Mean hemoglobin type change at 1 day, 1, 2 weeks, 1, 2, 3, and 6 months following infusion of the RSC into patients with beta-thalassemia when compared to baseline. At pre- (time point denoted Pre Apher) and just after apheresis (time point denoted Post Apher), patients received recent transfusions. From thereon, transfusion threshold was set at 7 g/dl.

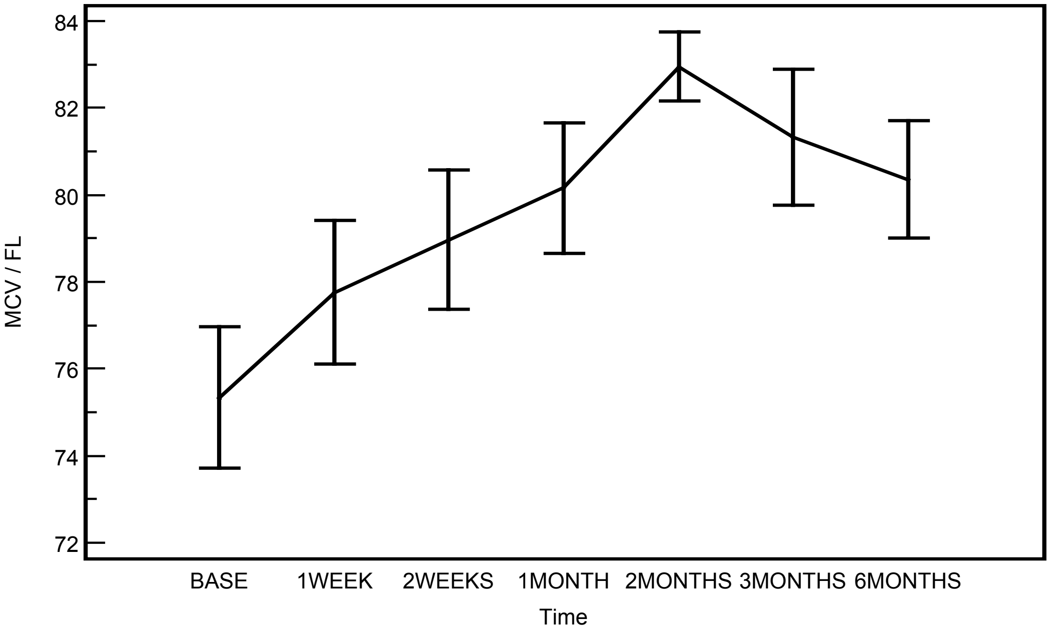

**FIGURE 5.** Increase in red blood cell MCV following infusion of the RSC into patients with beta-thalassemia. Artificial values at baseline due to recent transfusions.

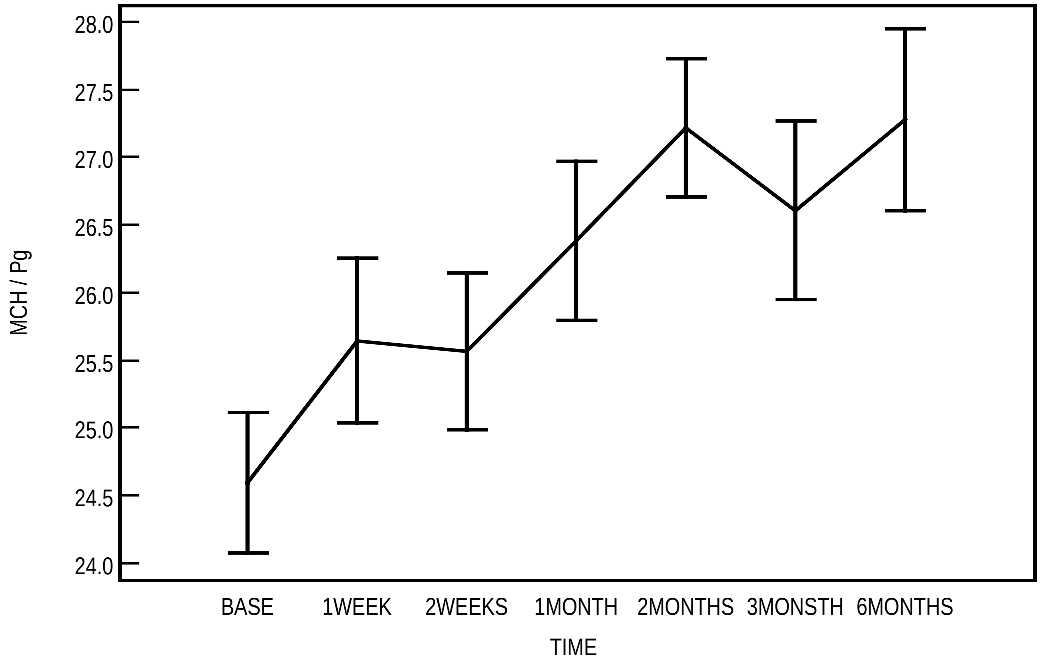

**FIGURE 6.** Increase in red blood cell MCH following infusion of the RSC into patients with beta-thalassemia. Artificial values at baseline due to recent transfusion.

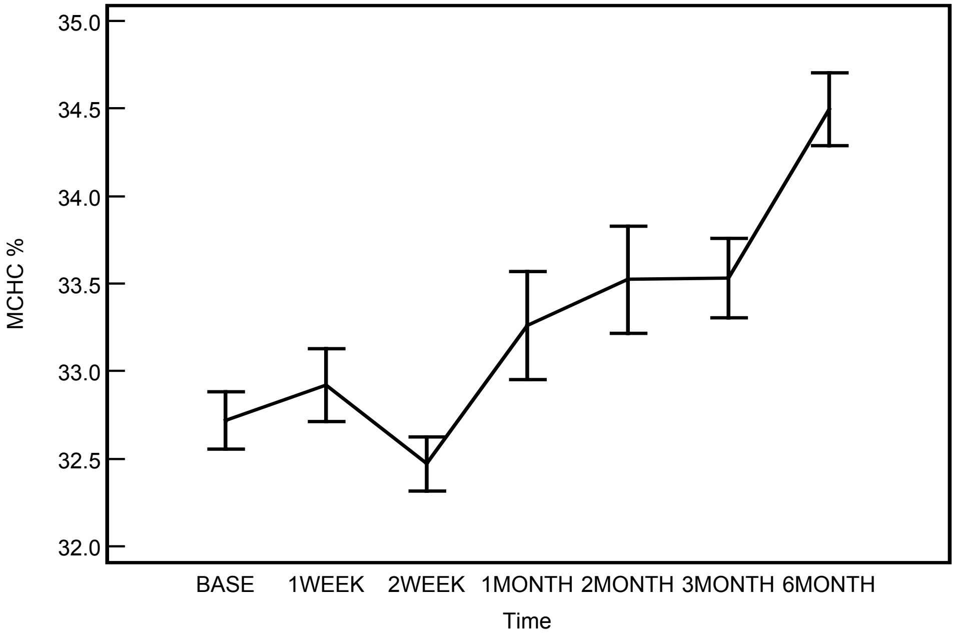

**FIGURE 7.** Increase in red blood cell MCHC following infusion of the RSC into patients with beta-thalassemia. Artificial values at baseline due to recent transfusion.

was accompanied by gradual decrease in MCV and MCH (from 72 fl and 23.1 Pg at baseline to 55.9 fl and 18.2 Pg, respectively, at 6 months), but not mean MCHC (patient 18). The decrease in MCV and MCH in this patient was mainly due to a significant increase in the number of red blood cells with much higher Hb E content Moreover, this patient who used to receive 3.5 units/90 days of packed red blood cell prior to infusion of the RSC is transfusion independent. On the other hand, the one patient (patient 21) with beta0-thalassemia/sickle cell anemia (Fig. 3) who is transfusion independent showed steady increase in Hb F (0.88 g/dl at baseline vs. 1 g/dl at 6 months) with a concomitant decrease in Hb S (8.4 g/dl at baseline vs. 7 g/dl at 6 months). This patient had no Hb A at all time points measured. Hb A2 was elevated in this patient at all times (0.52 g/dl at baseline vs. 0.48 g/dl at 6 months) while MCV, MCH, and MCHC increased (57.5 fl, 18.9 Pg, and 32.9% at baseline to 67.8 fl, 23.4 Pg, and 34.6%, respectively, at 6 months) steadily over the 6-month postinfusion period. Of note, this is the only patient that did not receive any blood transfusion pre- or postinfusion.

### Serum Ferritin, LDH, SGOT, SGPT, Creatinine, Bilirubin Direct and Indirect

In the absence of administering any chelating agents, mean serum ferritin (Table 3, Fig. 8) fell to statistically significant levels at 6 months ($p$ = 0.001) postinfusion (Fig. 8) of RSC when compared to baseline. Mean serum ferritin increased transiently to nonstatistically significant levels of 6029 ± 1255 ng/L ($p$ = 0.6) and 5008 ± 860 ng/ml ($p$ = 0.8), respectively, at 1 and 2 weeks postinfusion when compared to baseline (4463 ± 598 ng/ml).

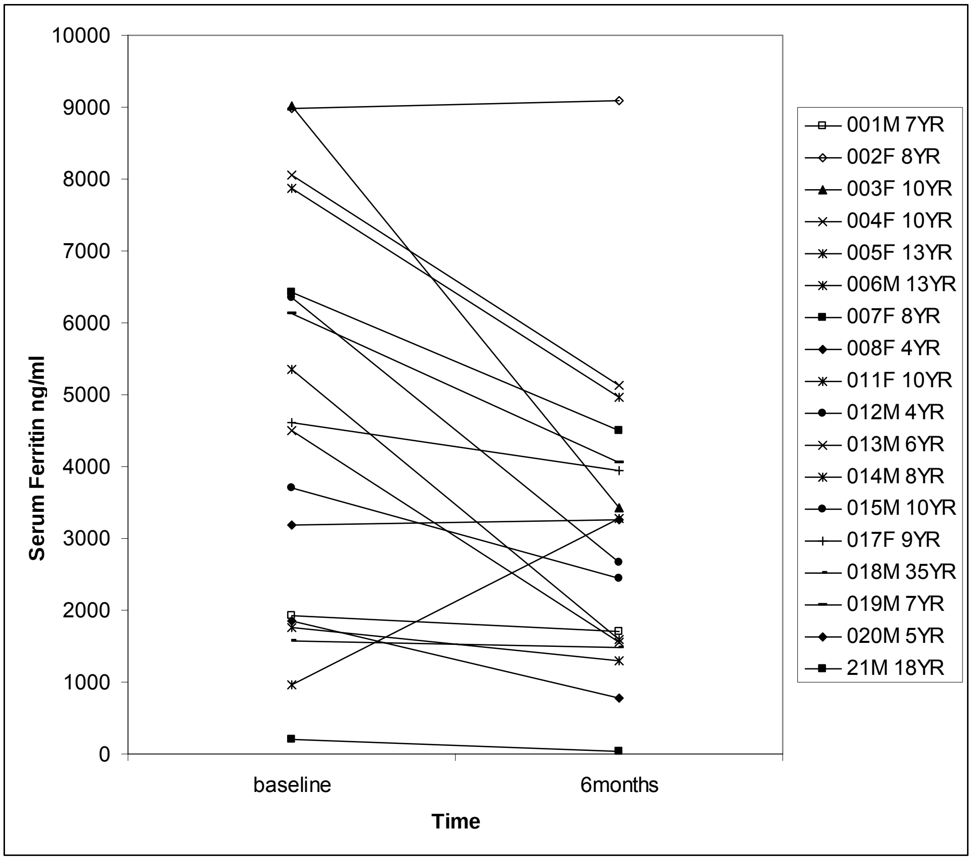

**FIGURE 8.** Decrease in serum ferritin at 6 months postinfusion of the RSC when compared to baseline. Each line style on the right denotes patient, sex, and age. No chelation therapy was given following infusion of the RSC.

The mean LDH (Table 3) rose to statistically significant levels at 1 ($p$ = 0.01), 2 ($p$ = 0.04), 3 ($p$ = 0.03), and 6 months ($p$ = 0.02) postinfusion when compared to baseline (Table 3).

The mean SGOT (Table 3) rose only to a statistically significant level at 1 week ($p$ = 0.001), 2 weeks ($p$ = 0.05), and 3 months ($p$ = 0.03) postinfusion when compared to baseline. The mean SGOT was not statistically different from baseline at, 1 ($p$ = 0.06), 2 ($p$ = 0.07), and 6 ($p$ = 0.15) months postinfusion (Table 3).

The mean SGPT (Table 3) rose to a statically significant level only at 1 week ($p$ = 0.03) postinfusion when compared to baseline. SGPT fell to a statistically nonsignificant level at 6 months postinfusion ($p$ = 0.15) when compared to baseline (Table 3).

Serum creatinine was within normal range at baseline and at 6 months postinfusion, but dropped slightly to statistically significant levels at 2 weeks, 1, 2, and 3 months when compared to baseline (Table 4).

**TABLE 4**
**Mean Neutrophils, Lymphocytes, and Platelets Before and After RSC Infusion into Beta-Thalassemia Patients**

| Test | Baseline | 1 Day | 1 Week | 2 Weeks | 1 Month | 2 Months | 3 Months | 6 Months |
|---|---|---|---|---|---|---|---|---|
| Neutrophils × $10^6$/ml | 3.6 ± 0.3 (1–6) N = 21 | 3.9 ± 0.5 (1–10) P = 0.1 N = 21 | 3.6 ± 0.4 (1–8) P = 0.6 N = 20 | 3.8 ± 0.3 (2–6) P = 0.6 N = 21 | 3.3 ± 0.4 (1–8) P = 0.7 N = 21 | 3.5 ± 0.6 (1–11) P = 0.6 N = 17 | 4.4 ± 0.6 (1–11) P = 0.5 N = 19 | 3.1 ± 0.3 (1–5) P = 0.03 N = 18 |
| Lymphocytes × $10^6$/ml | 3.6 ± 0.4 (1–9) N = 21 | 2.9 ± 0.5 (1–10) P = 0.01 N = 21 | 3.8 ± 0.4 (1–10) P = 0.6 N = 20 | 3.8 ± 0.4 (1–8) P = 0.3 N = 21 | 3.7 ± 0.5 (1–11) P = 1 N = 21 | 4.0 ± 0.6 (1–13) P = 0.9 N = 18 | 3.5 ± 0.6 (1–12) P = 0.2 N = 19 | 3.6 ± 0.6 (2–11) P = 0.3 N = 18 |
| Platelets × $10^6$/ml | 292 ± 32 (62–618) N = 21 | | 257 ± 24 (84–561) P = 0.3 N = 20 | 302 ± 34 (96–685) P = 0.8 N = 21 | 220 ± 22 (9–396) P = 0.03 N = 21 | 268 ± 38 (67–699) P = 0.3 N = 19 | 253 ± 20 (68–392) P = 0.04 N = 19 | 213 ± 26 (61–552) P = 0.01 N = 18 |
| Serum creatinine mg/dL | 0.4 ± 0.2 (0.3–0.6) N = 21 | | 0.4 ± 0.02 (0.2–0.6) N = 20 P = 0.5 | 0.3 ± 0.02 (0.2–0.5) N = 21 P = 0.03 | 0.3 ± 0.02 (0.2–0.5) N = 18 P = 0.02 | 0.3 ± 0.01 (0.2–0.4) N = 19 P = 0.0002 | 0.3 ± 0.02 (0.1–0.5) N = 18 P = 0.001 | 0.4 ± 0.02 (0.2–0.7) N = 19 P = 1 |
| Bilirubin direct mg/dL | 0.7 ± 0.1 (0.2–2) N = 21 | | 0.7 ± 0.1 (2–2.5) N = 20 P = 0.8 | 0.7 ± 0.1 (0.2–1.9 N = 21 P = 0.5 | 0.8 ± 0.1 (0.2–2) N = 18 P = 0.2 | 0.7 ± 0.1 (0.2–1.6) N = 19 P = 1 | 0.8 ± 0.2 (0.2–2.7) N = 19 P = 0.4 | 0.4 ± 0.1 (0–1.8) N = 19 P = 0.01 |
| Bilirubin Indirect mg/dL | 0.9 ± 0.1 (0.2–3.3) N = 21 | | 0.9 ± 0.1 (0.4–2.2) N = 20 P = 0.01 | 0.9 ± 0.1 (0.3–2) N = 21 P = 0.4 | 1 ± 0.1 (0.4–1) N = 18 P = 0.1 | 0.9 ± 0.1 (0.3–1.9) N = 19 P = 0.3 | 0.9 ± 0.2 (0.3–4.6) N = 19 P = 0.9 | 1 ± 0.2 (0.2–3) N = 19 P = 0.2 |

Bilirubin direct level was elevated at baseline and dropped to a statistically significant level at 6 months postinfusion when compared to baseline (Table 4).

Bilirubin indirect was at the same level at baseline, 2 weeks, 1, 2, 3, and 6 months postinfusion. However, it rose marginally to a statistically significant level at 1 week postinfusion when compared to baseline (Table 4)

### Neutrophils, Lymphocytes, and Platelets

The mean neutrophil (Table 4) count fell to a statistically significant level only at 6 months ($p$ = 0.03) postinfusion when compared to baseline (Table 4). The mean level of neutrophil count was more or less at the same level as baseline at 1 day, 1, 2 weeks, 1, 2, and 3 months postinfusion (Table 4).

The mean lymphocyte (Table 4) count fell to a statistically significant level at 1 day ($p = 0.01$) postinfusion when compared to baseline. The mean lymphocyte count at 1, 2 weeks, 1, 2, 3, and 6 months postinfusion was not statistically different to baseline.

The mean platelet (Table 4) count fell to significant levels at 1 ($p = 0.03$), 3 ($p = 0.04$), and 6 months ($p = 0.01$) postinfusion when compared to baseline level. The mean platelet count was not statistically different at 1, 2 weeks and 2 months postinfusion when compared to baseline.

### Bone Marrow, Karyotyping and G-Banding Analysis

Bone marrow smear and trephine section analysis around 2 weeks postinfusion in 18 thalassemic patients who gave consent, showed erythroid hyperplasia, active myelopoiesis with normal maturation, and differentiation and no abnormal cells. The myeloid to erythroid ratio was 1:2, and megakaryocytes and lymphocytes were present. Karyotyping and G-banding of peripheral blood samples of 18 patients were normal prior to and at around 3–6 months postinfusion.

### Echocardiogram and Bone Age Studies

Apart from 1 patient, 19 patients showed within the normal range echocardiogram results, following infusion of the RSC. In contrast, bone marrow age analysis was suboptimal for 20 thalassemic patients.

### DISCUSSION

Beta-thalassemia is found most commonly in the Mediterranean region, Africa, and Southeast Asia, presumably as an adaptive association to endemic malaria. The incidence may be as high as 10% in these areas[19]. The major causes of morbidity and mortality are anemia and iron overload[20]. The severe anemia resulting from this disease, if untreated, can result in high-output cardiac failure, while the intramedullary erythroid expansion may result in associated skeletal changes, such as cortical bone thinning. The long-term increase in red cell turnover causes hyperbilirubinemia and bilirubin-containing gallstones. Increased iron deposition resulting from multiple lifelong transfusions and enhanced iron absorption results in secondary iron overload[21]. This overload causes clinical problems similar to those observed with primary hemachromatosis[22], such as endocrine, liver, and cardiac dysfunctions. The manifestations of the disease may not be apparent until a complete switch from Hb F to Hb A synthesis occurs. This switch typically is completed by the sixth month after birth. Beta-thalassemia is caused by a genetic mutation in the beta-globin gene and many additional factors have been noted to influence the clinical manifestations of the disease. For example, the same mutations may have different clinical manifestations in different patients. One such factor is that patients with high Hb F concentration or hereditary persistence of Hb F syndrome have milder disease[23]. This latter finding has intensified efforts to identify Hb F-augmenting agents that have been shown to ameliorate beta-globin diseases such as sickle cell anemia[24] and some cases of beta-thalassemia[25]. In third-world countries where this disease is more prevalent, as well as perpetual[26], the complex socioeconomic factors make available treatments or a cure[27] beyond the reach of many young individuals.

Herein, the safety and efficacy of infusing 3-h autologous RSC into 21 patients with beta-thalassemia (19 were thalassemia major and only 2 were intermedia) was tested. No adverse side effects were noted postprocedure as determined by vital sign monitoring and various physical, laboratory, and clinical examinations, including echocardiogram (postinfusion only) analysis. All patients entering the trial were recently transfused, apart from one thalassemic/sickle cell anemia patient who did not receive any transfusion. Of note, the apheresis process resulted in a loss of about 1 g/L of total Hb (Fig. 4). Comparison of mean total Hb level post- (just prior to infusion) and preapheresis showed a significant statistical decrease (mean total Hb preapheresis = 9.3 ± 0.5 g/dl vs. mean total Hb postapheresis = 8.3 ± 0.5 g/dl, $p = 0.0003$). This loss in mean total Hb could also be due to the shearing effect of the double-lumen line on the rather fragile thalassemic red blood cells during apheresis. This loss was compensated by transfusing these patients with irradiated packed red blood cells prior to infusion of the RSC. The units of packed red blood cells transfused immediately following apheresis were not counted as part of the transfusion requirement at baseline. Karyotyping and G-banding of peripheral blood samples of 18 patients were normal prior to and at around 3–6 months postinfusion (data not shown). This observation indicates the stability of thalassemic white blood cells when exposed to the retrodifferentiation process. Bone marrow smear and trephine section analysis around 2 weeks postinfusion of RSC in 18 thalassemic patients who gave consent showed erythroid hyperplasia, active myelopoiesis with normal maturation, and differentiation with no abnormal cells present. The myeloid to erythroid ratio was 1:2 and megakaryocytes and lymphocytes were present. The mean blood transfusion requirement fell to a statistically significant level at 3 months (67%) and 6 months (50%) postinfusion (Table 3) when compared to baseline. Only 1 patient showed a significant increase in transfusion requirement (133%) at 3 months due to aplastic crisis caused by cholera infections during the postinfusion period. However, on intensive treatment of this patient, transfusion requirement fell to 50% at 6 months postinfusion when compared to baseline. His female sibling, who contracted the same infection and was treated, died at home just 2 months postinfusion. She was suffering from severe PAH as measured by echocardiogram analysis. Another patient died from blood-transfusion complications 3 months postinfusion, which was identified to be due to *E. coli*–contaminated blood.

The mean weight, height, and bone age of patients entering the trial were suboptimal for their age[28]. However, mean weight and height increased to statistically significant levels at 6 months postinfusion when compared to baseline (Table 3). This finding indicates that the RSC did not retard the growth of young thalassemic patients. However, it remains to be seen whether this increase in weight and height is of any significance when compared over the same time to age-matched control thalassemic patients, not receiving the RSC therapy.

The mean absolute level of Hb F rose steadily from 1.2 g/L at baseline to 2.9 g/L, 3.0 g/L, 3.9 g/dl, and 4.0 g/L at 1, 2, 3, and 6 months postinfusion, respectively (Table3, Fig. 4). This was accompanied by a significant decrease in the absolute level of mean Hb A. This statistically significant increase in mean Hb F absolute level was accompanied by a statistically significant increase in MCV, MCH, and MCHC (Table 3, Figs. 5, 6, and 7). This indicates that the infused RSC have resulted in the re-emergence of new red blood cells with higher red blood cell indices than donated adult red blood cells. This observation coupled with the significant increase in mean absolute Hb F levels (and the more or less constant levels of Hb A2, Fig. 4) suggests that the infused RSC are capable of fetal hematopoiesis and reontogeny. Fetal red blood cells possess inherently higher red blood cell indices[29] than normal adult red blood cells. Furthermore, Hb F levels have been noted to increase following cord blood stem cell transplantation and marks engraftment[30]. On the other hand, engraftment of the infused RSC is made more plausible in light of the fact that one thalassemia/Hb E patient (Fig. 3) had red blood cell indices typical of adult donated red blood cells at baseline, which became replaced following infusion by the rather smaller red blood cells typical of this Hb E variant (MCV, 72 fl, MCH 23.1 Pg, and MCHC, 32% at baseline vs. MCV, 55.9 fl, MCH, 18.2 Pg, and MCHC, 35% at 6 months). The same patient is transfusion independent 6 months postinfusion of RSC. Interestingly, Hb A in this patient steadily decreased to 0 g/L 3 months postinfusion and this was accompanied by a concomitant increase in Hb F (Fig. 2). The reason for achieving transfusion independence in this patient may be due to the well-known fact that subjects homozygous for Hb E variant[31] are asymptomatic, due to absence of the thalassemic Hb A or rather the aberrant beta-globin chain. This is the first time that Hb E switching has been noted, however, as to why the absolute Hb E concentration has increased significantly postinfusion is interesting indeed. This observation also suggest that both Hb alleles, Hb E and Hb A, are being differentially regulated in this patient due to the formation of a more stable hemoglobin structure comprising of Hb E and/or Hb $\gamma$ with the alpha-globin chains. In fact, this observation strengthens the notion that the RSC are also capable of contributing significantly towards adult hematopoiesis (at least replenishing about 8 g/dl times total blood volume worth of total Hb at 3–6 months in this particular patient) comprising a more stable Hb A variant such as Hb E (Fig. 2). On the other hand, in the absence of a more stable Hb A variant as is the case with most patients in this study (majority are beta0 where historical records are complete), the RSC have contributed more towards fetal rather than adult hematopoiesis, as exemplified by statistically significant decrease in mean Hb A and concomitant increase in Hb F when compared to baseline (Fig. 4). Interestingly, Hb A2, another form of Hb A, known to be intact in beta-thalassemia (elevated in beta-thalassemia intermedia), was noted to increase marginally only at 1 month following infusion of the RSC when compared to baseline. This increase, however, was not as sustainable as Hb F in these patients. Further evidence that supports the notion that infused RCS has contributed to fetal rather than adult erythropoiesis comes from the observation that the red blood indices of another patient with beta-thalassemia/Hb SC (Fig. 3) variant who never received any transfusion post or prior to this procedure have increased with time. The increase in MCV, MCH, MCHC (57.5 fl, 18.9 Pg, and 32.9% at baseline vs. 67.8 fl, 23.4 Pg, and 34.6%, respectively, at 6 months) and absolute Hb F were concomitant with decrease in Hb S in the absence of Hb A synthesis at all time points measured (Fig. 3). This patient already had high Hb A2 and Hb F levels at baseline, a finding consistent with a milder disease due to amelioration of Hb S hemoglobin polymerization in red blood cells[32]. Furthermore, despite being recently transfused, 74% of thalassemic patients entering the study had 0 g/L Hb F levels at baseline. On infusion, the number of patients exhibiting no Hb F synthesis fell to 53, 55, 47, and 40% 1, 2, 3, and 6 months postinfusion, respectively. This finding indicates the possibility of up-regulation of Hb F synthesis even in the absence of basal Hb F level. Low Hb F level in thalassemic patients at baseline is a predictor of a poor response to fetal augmenting agents and only effective in some patients with beta-thalassemia intermedia with high Hb F levels at baseline[33,34]. It would be very interesting to determine the frequency of apoptosis in the erythroid precursors in the bone marrow of thalassemic patients before and following infusion of the RSC, since decreased frequency of programmed erythroblast death and general improvement of the disease condition has been noted, in response to treatment with Hb F switching agent such as butyrate[35].

Even more interesting is the concurrent finding that serum ferritin[36,37] fell to statistically significant levels at 6 months postinfusion (Table 3, Fig. 8) of the RCS when compared to baseline. This observation is extremely hard to explain in the absence of receiving any chelation therapy[38]. However, one explanation to the significant fall may be due to the decrease in blood transfusion requirement[6]. Alternatively, the decrease in serum ferritin could be due to mobilization of tissue ferritin into the periphery and its subsequent reutilization in driving a novel ongoing juvenile or adult erythropoiesis[39]. This transient mobilization and specific reutilization of serum ferritin in hemoglobin synthesis may be explained by the statistically significant mean increase in serum ferritin at 1 ($p$ = 0.001) and 2 ($p$ =0 .001) weeks postinfusion when compared to 6 months postinfusion of RCS. This transient increase in serum ferritin appears to be proceeded by a statistically significant increase in mean LDH at 1, 2, 3, and 6 months postinfusion when compared to baseline (Table 3). This lagging profile of the increases in LDH and serum ferritin, with the gradual increase in Hb F synthesis, may reflect the release of sequestered tissue ferritin from damaged tissue and/or old and effete thalassemic red blood cells for the purpose of regeneration of new Hb F-containing red blood cells or otherwise more stable Hb form. Of note, serum ferritin did also fall to significant levels 6 months postinfusion of RSC in most of patients that did not exhibit any Hb F synthesis. However when Hb F expression was investigated in these patients by immunostaining and flow cytometry, Hb F expression was pan-cellular[40] in the red blood cells of the majority. Yet these patients exhibited different Hb F levels when measured by HPLC (data not shown). This may reflect the difference in the sensitivity between these two assays. Whether the significant decrease in serum ferritin at 6 months post RSC has resulted in actual decrease in iron overload remains to be seen. However, serum ferritin is a biomarker widely used to monitor iron overload and has been noted to correlate with liver iron[41] concentration and improvement in heart function in beta-thalassemia major[42]. Of note, visual analysis of stool and urine samples of these patients following infusion of the RSC showed extreme dark-brown coloration that could have been due to excreted iron and, therefore, may explain the significant drop in serum ferritin.

As to why and how RSC engrafted thalassemic patients in the absence of marrow ablation such as, chemo or radiotherapy remains at large. However one possibility is that the autologous RSC may be more primitive than sister endogenous stem cells[43] and, therefore, have a selective advantage due to their capability of embarking on a fetal rather than adult program of red blood cell development; the latter of which is being continually and prematurely terminated by the spleen. Alternatively, ineffective erythropoiesis in these patients may facilitate the takeover of endogenous progenitors by the more primitive RSC due to expression of a more stable hemoglobin configuration. Interestingly, a significant proportion of thalassemic RSC did express CD34 in the absence of CD38 (Fig. 1). This phenotype is typical of more primitive and long-lived hematopoietic stem cells[44,45]. In fact, autologous RSC have been able to engraft the bone marrow of acquired severe aplastic anemia (manuscript in preparation) patients and have induced transfusion independence for nearly 2 years now with significant Hb F switching. It remains to be seen whether a state of bone marrow suppression prior to infusion of the RSC is a perquisite for more persistent and enhanced Hb F expression in the beta-hemoglobinopathies disorders.

In general, liver enzymes (Table 3) were more or less the same at baseline and postinfusion. However, mean SGOT rose to a significant level at 1 week and 3 months postinfusion compared to baseline. Mean SGPT rose only to a significant level at 1 week when compared to baseline and fell to a nonstatistically significant level at 6 months. As to why this transient rise in mean SGOT and SGPT occurred remains unclear. One possibility is that the rise in liver enzymes at 1 week postinfusion may have directly exaggerated the transient, nonstatistically significant rise in serum ferritin at 1 and 2 weeks postinfusion, since the latter can also act as an acute phase reactant[46]. The mean level of neutrophils, lymphocytes, and platelets (Table 4) fluctuated with time. Only at 6 months, the mean level of neutrophils fell to a statistically significant level when compared to baseline, whereas lymphocyte transiently fell to a statistically significant level at 1 day postinfusion when compared to baseline. On the other hand, platelet count fell to statistically significant levels at 1, 3, and 6 months postinfusion when compared to base. As to why these minor changes still remain within normal ranges in neutrophils and platelet counts postinfusion remains to be seen. However, skewed hematopoiesis towards the erythroid rather than the myeloid lineage may be the driving force, since the latter is more or less intact in nonsplenecotomized thalassemic patients[47]. Alternatively, patients suffering from thalassemia have high nucleated red blood cells and platelet counts, which could have exaggerated baseline values since some hematological counters cannot accurately differentiate between microcytic red blood cell or platelets, or between nucleated red blood cells and various white blood cell types.

Bilriubin direct, which is an indicator of bile obstruction and liver damage, fell to a statistically significant level at 6 months postinfusion when compared to baseline. On the other hand, the indicator of hemolysis, bilirubin indirect, was at the same level before and following RSC infusion when compared to baseline (Table 4). Bilirubin indirect rose slightly to a statistically significant level at 1 week postinfusion when compared to baseline (Table 4). This mild elevation in bilirubin indirect could have been due to hemolysis effect of apheresis on the rather fragile thalassemic red blood cells.

Serum creatinine fell to statistically significant levels at 2 weeks, 1, 2, and 3 months postinfusion when compared to baseline, but remained within normal range.

It should be noted that receiving a blood transfusion is always based on the provision of donors (usually provided by patient's relatives or willing donors) to replenish blood supply in local blood banks. Most relatives are either affected by thalassemia or unable to donate blood due to recent donation. Most importantly, potential volunteers do not appreciate the benefit of blood donation and some are HCV positive. This scenario has put a constraint to include age-matched thalassemic controls in this study. Quantitative assessment of liver and spleen size as well as morphological imaging before and following infusion of the RSC would have been important in explaining the significant decrease in the biomarker of iron overload, serum ferritin, in these thalassemic patients. Apart from one patient, echocardiogram analysis of all patients showed normal value 3 months postinfusion of the RSC; however, in the absence of baseline values, it remains to be seen whether the infusion of the RSC did actually improve heart function in these patients. Finally, observation over the same time of 12 patients with nonhematological disorders, who received the RSC, did not show any significant change in serum ferritin, Hb F, or red blood indices when compared to baseline. However, these values were not measured consistently in all patients as in the thalassemic patients due to patients' noncompliance to this frequent testing. This finding here may have confirmed the notion that RSC repair mechanism is tissue-injury specific.

### CONCLUSION

In conclusion, the single-dose infusion of the autologous RSC into patients with beta-thalassemia major has resulted in a reduction in blood transfusion requirement and improved red blood cell morphology, hemoglobin content, and concentration. Furthermore, the biomarker of iron overload, serum ferritin, fell to a statistically significant level at 6 months postinfusion when compared to baseline. This novel regenerative approach towards ameliorating a genetic disorder, though most probably transitory, is profound and may enhance compliance of young thalassemic patients. Most importantly, this procedure may benefit patients that have no HLA-identical or genetically unaffected siblings[48].

### ACKNOWLEDGMENTS

We would like to thank Mr. Tariq Mumtaz and Mr. Abdullah Hussain Saeed Haroon of Healthcare Solutions International (Pvt) Limited for sponsoring the study. We are grateful to Prof. Dr. Ata-Ur-Rahman, Federal Minister and Chairman of the Higher Education Commission of Pakistan for constructive discussion. We are indebted to Mr. Alpha Nasim David, Laboratory Technician and Mr. Mazharul Islam Siddiqui for patient assistance. We would also like to thank Dr. R. Arif Rahim, Medical Director, and Dr. Aslam Pervez, Patient Services Administrator of the Orthopedic and Medical Institute. We are immensely grateful to the Fatimid Foundation for the provision of blood products, Mr. Muhammad Ilyas Khan for performing the apheresis and Mr. Muhammad Akhter Anwer for the statistical analysis. Finally, we would like to thank the patients and their families who participated in this study.
